# PDGFR-Targeted Ruxolitinib Liposomes Modulate Fibroblast-Associated Colorectal Cancer–Stroma Interactions, Reducing Invasion and Enhancing Chemosensitivity

**DOI:** 10.1021/acsomega.6c03367

**Published:** 2026-08-25

**Authors:** Paweena Dana, Anukul Taweechaipaisankul, Onuma Phoraksa, Monthira Rattanatayarom, Walailuk Chonniyom, Primana Punnakitikashem, Prattana Tanyapanyachon, Nattika Saengkrit

**Affiliations:** † 61191 National Nanotechnology Center (NANOTEC), National Science and Technology Development Agency (NSTDA), 111 Thailand Science Park, Phahonyothin Road, Khlong Nueng, Khlong Luang, Pathum Thani 12120, Thailand; ‡ Department of Biology, Faculty of Science, 133942 Chulalongkorn University, Bangkok 10330, Thailand; § Department of Biochemistry, Faculty of Medicine, Siriraj Hospital, 65106 Mahidol University, 2 Wanglang Road, Bangkoknoi, Bangkok 10700, Thailand

## Abstract

Ruxolitinib (Rux) is a tyrosine kinase inhibitor targeting the Janus kinase (JAK) and signal transducer and activator of transcription (STAT) pathways and is clinically used for the treatment of myelofibrosis. In this study, a nanodelivery approach was developed to investigate the effects of Rux on fibroblast-associated tumor–stroma interactions in colorectal cancer. Platelet-derived growth factor receptor (PDGFR)-targeted, Rux-loaded liposomes (Lip/Rux/PDGFR) were prepared using a lipid thin-film hydration method and characterized by dynamic light scattering. The average hydrodynamic diameters of Lip/Rux and Lip/Rux/PDGFR were 186.2 ± 1.2 nm and 178.6 ± 2.0 nm, respectively, with slightly negative surface charges. Transmission electron microscopy confirmed the spherical morphology. Antiproliferative activity was evaluated in HT-29 colorectal cancer cells using MTT and three-dimensional tumor spheroid assays. Lip/Rux and Lip/Rux/PDGFR reduced HT-29 cell viability compared with free Rux and Lip/PDGFR controls. To assess stromal-associated effects, activated fibroblasts were treated and subsequently evaluated in coculture and 3D spheroid invasion models. Treatment of activated fibroblasts with Lip/Rux/PDGFR was associated with a greater reduction in HT-29 cell invasion than free Rux and Lip/PDGFR. Furthermore, pretreatment of activated fibroblasts with Lip/Rux/PDGFR was associated with improved 5-FU responsiveness in cocultured HT-29 spheroids, suggesting a reduction in fibroblast-associated chemoprotective effects. Taken together, these findings suggest that PDGFR-targeted liposomal delivery of Rux may affect fibroblast-associated tumor–stroma interactions, which could contribute to reduced colorectal cancer cell invasion and enhanced sensitivity to chemotherapy. Further studies are needed to clarify the underlying mechanisms and determine whether these effects are mediated through alterations in activated fibroblast functions.

## Graphical abstract

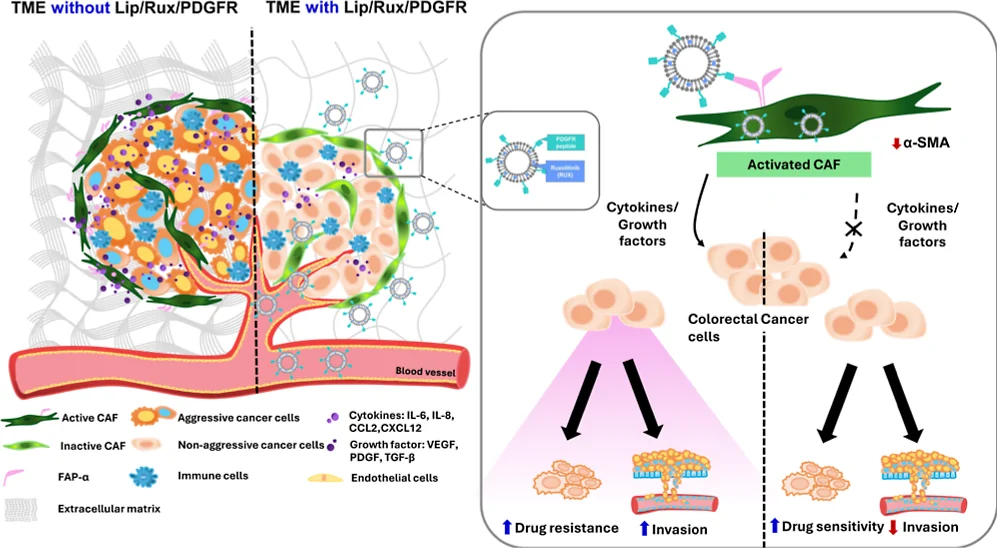

## 1 Introduction

Colorectal cancer (CRC) is the third most common type of cancer worldwide^1,2^ and is a malignancy of the colon and rectum. The stage of the CRC diagnosis defines the survival rate of the patients.^3^ The earlier the CRC is detected, the higher the curable rate by surgery and subsequent medications. However, the early diagnosis alone does not guarantee a positive prognosis, as certain characteristics of CRC could lead to treatment failures. CRC often results in liver metastasis and a high rate of tumor recurrence and even exhibits drug resistance.^1,4,5^ Thus, to increase the CRC patients’ survival rate, a study on inhibiting CRC tumor metastasis and drug resistance is urgently needed.

The desmoplastic reaction, characterized by dense fibrosis and stromal cell accumulation, is a hallmark of several solid tumors, including breast, prostate, pancreatic, cholangiocarcinoma, and colorectal cancer (CRC). Within the tumor microenvironment (TME), stromal cells are divided into cellular and noncellular compartments. The cellular compartment consists of cancer-associated fibroblasts (CAFs), endothelial cells, and inflammatory cells, while the noncellular compartment is composed of the extracellular matrix (ECM), which includes collagen, proteoglycans, fibronectin, growth factors, and cytokines. Together, these elements of the TME foster tumor progression, metastasis, and resistance to therapies such as chemotherapy and radiotherapy.^6–8^

Among stromal cells, CAFs play a crucial role in tumor development.^9,10^ Normally, fibroblasts can be activated by stimuli such as growth factors, cytokines, and cellular stress, transforming into CAFs.^10–12^ Transforming growth factor beta 1 (hTGF-β1), secreted by both stromal and tumor cells, enhances fibroblast activation into CAFs and promotes tumor migration and metastasis. CAFs are typically identified by the expression of biomarkers including α-smooth muscle actin (α-SMA), fibroblast activation protein (FAP), interleukin-6 (IL-6), monocyte chemoattractant protein-1 (MCP-1), and platelet-derived growth factor (PDGF) receptor.^10^ In CRC tissues, immunohistochemical analysis of stromal FAP has been used to assess CAF accumulation. Notably, high FAP expression correlates with aggressive cancer phenotypes, increased metastasis, and reduced patient survival, compared to cases with low FAP expression.^13^

Within the TME, CAFs play multiple roles in enhancing tumor growth and metastasis. They achieve this through the secretion of growth factors, cytokines, chemokines, and ECM proteins, which collectively activate diverse signaling pathways that enhance cell proliferation and metastatic potential, ultimately contributing to poor patient outcomes.^6,7,14^

As cancer progresses, CAFs also mediate mechanical remodeling of the stromal ECM. By secreting ECM proteins, they generate a dense fibrotic stroma and elevate interstitial fluid pressure. These changes not only reinforce the tumor’s structural rigidity but also significantly impair the penetration and delivery of chemotherapeutic agents to malignant cells, thereby reducing treatment efficacy.^15,16^

CAFs, as a dominant component of the TME, have appeared as promising therapeutic targets. This strategy is based on reprogramming or normalizing the TME to overcome physiological barriers that hinder drug delivery.^15–20^ Early approaches focused on CAF depletion, but studies in mice revealed systemic side effects.^21^ Consequently, reprogramming CAFs’ functions and deprogramming activated fibroblasts are now considered more attractive and clinically practical strategies.^22^

In targeted cancer therapy, monoclonal antibodies and small-molecule inhibitors directed against key regulatory factors in CAFs biology have been evaluated in both preclinical and clinical settings.^23,24^ The challenging stromal barrier created by CAF activity remains a major obstacle to effective drug penetration. Thus, disrupting this barrier represents a convincing strategy for enhancing therapeutic delivery.

One innovative approach involves the design of nanoparticles that specifically target CAFs, aiming to diminish their activation and remodel the TME. Such CAF-targeting nanoparticles can reshape stromal architecture, reduce drug resistance, and improve the efficiency of drug delivery to tumor cells. Several studies have reported that these nanoparticles achieve antitumor effects either by eliminating CAFs or by modifying their functions.^25–27^

Ruxolitinib is a tyrosine kinase inhibitor that specifically targets the Janus kinase (JAK) and signal transducer and activator of the transcription (STAT) pathway. It is a potent and selective oral inhibitor of JAK1 and JAK2. In 2011, the U.S. Food and Drug Administration (FDA) approved ruxolitinib for the treatment of myelofibrosis, and it has since been applied in managing polycythemia vera and steroid-refractory graft-versus-host disease following allogeneic stem-cell transplantation.^28^

Not only that, but Ruxolitinib has also been investigated in the context of solid tumors. In syngeneic ovarian cancer models, studies have examined the combined impact of ruxolitinib and paclitaxel (Taxol) therapy on both tumor cell biology and immune cell function, highlighting its potential role in modulating the tumor microenvironment.^29^

In this study, we propose a novel therapeutic strategy for CRC by targeting and inactivating activated fibroblasts within the TME. To achieve this, we developed a ruxolitinib-loaded liposomal formulation conjugated with PDGFR ligands on its surface (Lip/Rux/PDGFR), enabling specific recognition of activated fibroblasts. This targeted nanocarrier offers promising potential as a drug-delivery system.

The specificity of Lip/Rux/PDGFR enhanced cellular uptake and effectively reprogrammed activated fibroblasts to a normal fibroblast-like phenotype. By inactivating fibroblasts, the formulation suppressed HT-29 colorectal cancer aggressiveness, as evidenced by reduced tumor spheroid growth and invasion while simultaneously increasing the sensitivity of HT-29 cells to the chemotherapeutic agent 5-fluorouracil (5-FU).

Overall, this study introduces a novel stromal-targeted therapeutic approach to CRC. By reshaping the TME through CAF inactivation, Lip/Rux/PDGFR offers a promising platform that may be extended to other stroma-rich cancers, paving the way for more effective combination therapies.

## 2 Materials and Methods

### 2.1 Materials

Dulbecco’s modified Eagle’s medium (DMEM) (cat. no. 12800017), penicillin–streptomycin–glutamine (100X) (cat. no. 10378016), trypsin-EDTA (cat. no. 25200056), fetal bovine serum (FBS) (cat. no. 10270106), and IL-6 enzyme-linked immunosorbent assay (ELISA) (cat. no. 88-7066-22) were purchased from GIBCO Invitrogen (NY, USA). Matrigel Growth Factor-Reduced (cat. no. 354230) 96-well plate (cat. no. 3590), 96-well round-bottom ultralow-attachment plates (cat. no. 7007), and 8.0 μm pore size insert (cat. no. 3422) were purchased from Corning (NY, USA). For the MTT assay, 3-(4,5-dimethylthiazol-2-yl)-2,5-diphenyltetrazolium bromide (cat. no. 475989) was purchased from EDM Millipore Corp. (MA, USA). Ruxolitinib (cat. no. S1378) and dimethyl sulfoxide (DMSO) (cat. no. 102952) were purchased from Merck Millipore (Sigma-Aldrich; Merck Millipore, Darmstadt, Germany). Soybean lecithin (soya phosphatidylcholine; PC) was purchased from Degussa (Hamburg, Germany). Cholesterol was purchased from Wako Fujifilm (Osaka, Japan). Sulforhodamine B (cat. no. S1402-1G) was purchased from Sigma-Aldrich (Darmstadt, Germany). A two-hundred-mesh carbon-coated copper grid (cat. no. EMS200-Cu) was purchased from Electron Microscopy Sciences (Hatfield, PA, USA). A dialysis bag with a molecular weight cutoff of 100 kDa was purchased from CelluSep (Seguin, TX, USA). The TRIzol reagent (cat. no. 15596026) was purchased from Thermo Fisher Scientific (Waltham, MA, USA). iScript Reverse Transcription Kits (cat. no. 1708840) were purchased from Bio-Rad Laboratories (Hercules, CA, USA). Luna Universal qPCR (cat. no. M3003L) was purchased from New England Biolabs Inc. (Ipswich, MA, USA). Platelet-derived growth factor receptor (PDGFR)-specific peptide was purchased from GenScript Biotech Corporation (Nanjing, China).

### 2.2 Methods

#### 2.2.1 Cell Culture

The human colorectal adenocarcinoma cell line (HT-29; ATCC HTB-38) and the human normal lung fibroblast cell line (MRC-5; ATCC CCL-171) were cultured in DMEM supplemented with 10% FBS, 0.1 mM nonessential amino acids (100 μg/mL l-glutamine), 100 μg/mL streptomycin, and 100 U/mL penicillin, with the incubation condition of 37 °C with a humidified atmosphere containing 5% CO_2_.

#### 2.2.2 Formulation of PDGFR-Specific Peptide-Conjugated Ruxolitinib-Loaded Liposome (Lip/Rux/PDGFR)

The Lip/Rux/PDGFR was prepared using the thin-film method. Briefly, soybean lecithin was mixed with cholesterol in a molar ratio of 8:1, along with 0.1% phosphatidyl ethanolamine polyethylene glycol (PEG–PE) and MPB-PE (linker), using chloroform–methanol (2:1 v/v, 10 mL) as the solvent. Ruxolitinib (8 mg/in 2 mL methanol) was added to the mixture. The solvents were then removed using a rotary evaporator, obtaining a thin film of ruxolitinib-loaded liposome (Lip/Rux/-). To obtain the conjugated liposome (Lip/Rux/PDGFR), the PDGFR-specific peptide was conjugated to Lip/Rux/- by adding the peptide and stirring at 4 °C overnight. The excess PDGFR-specific peptide residues were removed by ultracentrifugation at 80,000 rpm for 2 h.

The encapsulation efficiency of ruxolitinib was determined via HPLC. Briefly, ruxolitinib liposomes were added to the ultrafiltration tube (Amicon Centrifugal Filter Unit, 100 kDa molecular weight cutoff) and centrifuged at 7500 RCF for 3 h, twice. Next, samples in the tube were collected to determine ruxolitinib concentrations by HPLC with a C18 column. The mobile phase consisted of acetonitrile and water at a ratio of 55:45 (v/v) with a flow rate of 1.2 mL/min. The detection was measured at 310 nm. The encapsulation efficiency was calculated using the following equation

Encapsulationefficiency(%)=(Ruxolitinib/initialRuxolitinib)×100

#### 2.2.3 Physicochemical Characterization of (Lip/Rux/PDGFR)

The morphologies of empty liposomes (Lip/–/−), Lip/Rux/-, LIP/-/PDGFR, and Lip/Rux/PDGFR were observed using transmission electron microscopy (TEM). 5 μL of the sample was dropped on a carbon-coated grid and left for 10 s, and then the excess was removed using filter paper. When the grid was dried, 5 μL of 0.5% phosphotungstic acid was dropped to obtain the negative staining. The prepared grid was observed under TEM (Hitachi HT7800, Hitachi, Japan) with 80 kV at magnifications of 50,000× and 100,000×.

The samples of (Lip/–/−), Lip/Rux/-, LIP/-/PDGFR, and Lip/Rux/PDGFR were diluted 50-fold with filtered distilled water to 1 mL. The diluted sample was measured for size, polydispersity index (PDI), and zeta potential using dynamic light scattering (DLS) at 25 °C.

#### 2.2.4 *In Vitro* Drug Release Study of Lip/Rux/-

0.5 mL of Lip/Rux/- and free Ruxolitinib were added into dialysis bags with a molecular weight cutoff of 100 kDa. The dialysis bags were dialyzed against phosphate-buffered saline (PBS), pH 7.4, with 0.5% TWEEN 80 (10 mL) at 37 °C under continuous stirring of 180 rpm.^30,31^ At different time points such as 0, 1, 2, 4, 6, 24, and 48 h, 1 mL of PBS was collected from the system and replaced by an equivalent volume of fresh PBS. The collected PBS was then analyzed with high-performance liquid chromatography (HPLC) to determine the Ruxolitinib concentration.^32^ The C18 column was used with a methanol–water solvent mixture (65:35) as a mobile phase, and the Ruxolitinib was detected at the wavelength of 295 nm.

#### 2.2.5 MTT Assay for Cytotoxicity Evaluation of Lip/Rux/PDGFR

The cytotoxicity of Lip/Rux on MRC-5-activated MRC-5 and HT-29 cell lines was determined by an MTT tetrazolium reduction assay. The cells were seeded individually onto 96-well plates with cell densities of 4 × 10^3^ and 1 × 10^4^ cells/100 μL/well, respectively. The seeded cells were cultured overnight in complete DMEM at 37 °C and 5% CO_2_. Then, the cells were treated with free Rux (Rux), empty liposome (Lip/–/−), Lip/Rux/-, LIP/-/PDGFR, and Lip/Rux/PDGFR with the range of 0–100 μM Ruxolitinib, in complete DMEM, and cultured for 48 and 72 h. After 48 and 72 h, the final concentration of 0.5 mg/mL of MTT was added to each well, and the plates were further incubated at 37 °C for 4 h. After 4 h, the MTT solution was removed and replaced by 100 μL of DMSO to dissolve the formazan crystals. The microplate reader (Spectra-MAX, CA, USA) at a wavelength of 570 nm was used to measure the absorbance. Each experiment was performed in triplicate, and three independent experiments were performed.

#### 2.2.6 Activation of Fibroblasts Using CRC-Derived Conditioned Medium

The activation of a normal human fibroblast, in this experiment, MRC-5, by culturing MRC-5 in HT-29’s conditioned media (CM), was done to mimic the CAF condition. 2 × 10^5^ cells/well of MRC-5, normal lung fibroblast cells, were seeded onto a 6-well plate. After 24 h, the media was replaced with 50% HT-29’s CM in complete DMEM. And after another 24 h, the media was replaced with serum-free DMEM and incubated for 24 h. To confirm the activation, α-smooth muscle actin (α-SMA) was determined, as α-SMA is overexpressed on the CAF surface and is one of the distinguishing characteristics between CAFs and normal fibroblasts.^10^ Along with the detection of PDGFR. The detection was done by using immunofluorescent labeling.

#### 2.2.7 Inactivation of Activated Fibroblasts Using Lip/Rux/PDGFR

The activated MRC-5 (Act-MRC-5), which was obtained from Section 2.2.6, was used to investigate the reprogramming ability of Lip/Rux/PDGFR. The concentrations of Ruxolitinib of 5 and 10 μM from free Rux, Lip/–/–, Lip/Rux/-, LIP/-/PDGFR, or Lip/Rux/PDGFR were treated with the Act-MRC-5 for 72 h. After 72 h of incubation, the media were replaced with serum-free media for another 24 h. Then the conditioned medium was harvested and centrifuged at 2,000 x *g*, 4 °C for 5 min to remove debris cells. The expression of α-SMA, a key activated fibroblast marker, was evaluated using immunofluorescent staining.

#### 2.2.8 Effect of Lip/Rux/PDGFR-Treated Act-MRC-5 on 3D Tumor Spheroid Cell Viability of CRC Cells

The indirect coculture 3D spheroid of HT-29 treated with Rux, Lip/Rux/-, LIP/-/PDGFR, or Lip/Rux/PDGFR-treated Act-MRC-5-derived CM. Briefly, 100 μL of HT-29 with a cell density of 1500 cells/well was seeded into 96-well round-bottom ultralow-attachment plates and incubated for 3 days to form spheroids. The CM of Rux, Lip/Rux/-, LIP/-/PDGFR, or Lip/Rux/PDGFR-treated Act-MRC-5 was then added to the spheroids and incubated for another 4 days. The Olympus IX50 inverted microscope (Olympus, Tokyo, Japan) was used to take bright-field images of HT-29 tumor spheroids treated with the samples.

To assess the impact of inactivating Act-MRC-5 using Lip/Rux/PDGFR on CRC cells’ sensitivity to chemotherapy, Act-MRC-5 cells were treated with Rux, Lip/Rux/–, Lip/–/PDGFR, or Lip/Rux/PDGFR with the Ruxolitinib concentration of 5 or 10 μM for 72 h. After treatment, cells were washed and incubated in serum-free medium for 24 h. Then, the CM was harvested for coculture experiments with HT-29 spheroids (Act-MRC-5-derived CM).

To further evaluate the effect of fibroblast inactivation on 5-fluorouracil (5-FU) sensitivity, HT-29 spheroids (3 days old) were treated with Act-MRC-5-derived CMs (as mentioned above), and the treatment was combined with 5-FU at concentrations of 1 or 5 μM. After 96 h of incubation, spheroid morphology was observed under an Olympus IX50 inverted microscope (Olympus, Tokyo, Japan), along with the spheroid volume quantified using ImageJ software (Bethesda, MD, USA), and cell viability was assessed with the 3D ATPlite kit (PerkinElmer). All experiments were performed in triplicate wells.

#### 2.2.9 Spheroid Invasion Assay

The three-day-cultured HT-29 spheroids were collected and embedded into 100 μL of 1:32 diluted Matrigel in cold serum-free DMEM in an ultralow-attachment plate. The Matrigel was solidified by incubating at 37 °C for 1 h. After 1 h, the Act-MRC-5-derived CM (Rux, Lip/-/PDGFR, or Lip/Rux/PDGFR-treated Act-MRC-5 CM) was added to the well. The treated spheroids were observed and captured under an Olympus IX50 inverted microscope (Olympus, Tokyo, Japan) at 0, 24, 48, 72, and 96 h. The spheroid-invasion area was quantified compared to the control, which was a spheroid treated with complete DMEM, using ImageJ software. The experiment was performed in triplicate.

#### 2.2.10 Statistical Analysis

The data are presented as mean ± standard deviation. Significant differences between the experimental groups were assessed using Student’s *t*-test or one-way ANOVA followed by Tukey’s multiple comparisons, with a *P*-value of less than 0.05 regarded as statistically significant. All statistical analyses were conducted using SPSS version 17.0 (SPSS Inc., Chicago, IL, USA).

## 3 Results and Discussion

### 3.1 Morphology and Physicochemical Characteristics of Lip/Rux/PDGFR

The Lip/Rux/PDGFR was prepared using thin-film hydration with the lipid components, as shown in Figure 1A. A transmission electron microscope was employed to observe the sizes and morphologies of Lip/Rux/PDGFR, Lip/Rux/-, and Lip/–/–. All three exhibited a spherical shape with a diameter of approximately 170–190 nm, as observed under magnifications of 50,000× and 100,000× (Figure 1B). The TEM images indicated that the diameters of Lip/–/–, Lip/Rux/-, Lip/-/PDGFR, and Lip/Rux/PDGFR were 100–200 nm, which are preferentially extravasated into the tumor tissues via the EPR effect. The physicochemical characteristics of Lip/–/–, Lip/Rux/-, Lip/-/PDGFR, and Lip/Rux/PDGFR were determined using DLS. As indicated in Table 1, the diameters and zeta potentials of Lip/–/–, Lip/Rux/-, Lip/-/PDGFR, and Lip/Rux/PDGFR were evaluated using DLS. The average diameters of the Lip/–/–, Lip/Rux/-, Lip/-/PDGFR, and Lip/Rux/PDGFR nanoparticles were 196.47 ± 2.95 nm, 176.43 ± 6.83 nm, 196.03 ± 5.84 nm, and 175.70 ± 2.55 nm, respectively. The average zeta potentials of Lip/–/–, Lip/Rux/-, Lip/-/PDGFR, and Lip/Rux/PDGFR nanoparticles were −7.57 ± 0.27 mV, −3.31 ± 0.69 mV, −9.30 ± 0.39 mV, and −2.19 ± 0.52 mV, respectively. Lip/–/–, Lip/Rux/-, Lip/-/PDGFR, and Lip/Rux/PDGFR nanoparticles were shown to be homogeneous, as demonstrated by a narrow size distribution with a PDI value of 0.11 ± 0.03, 0.08 ± 0.05, 0.16 ± 0.04, and 0.11 ± 0.02, respectively. This result agrees well with the low PDI of liposome nanoparticles (<0.2) found previously.^33^

**Figure 1:**
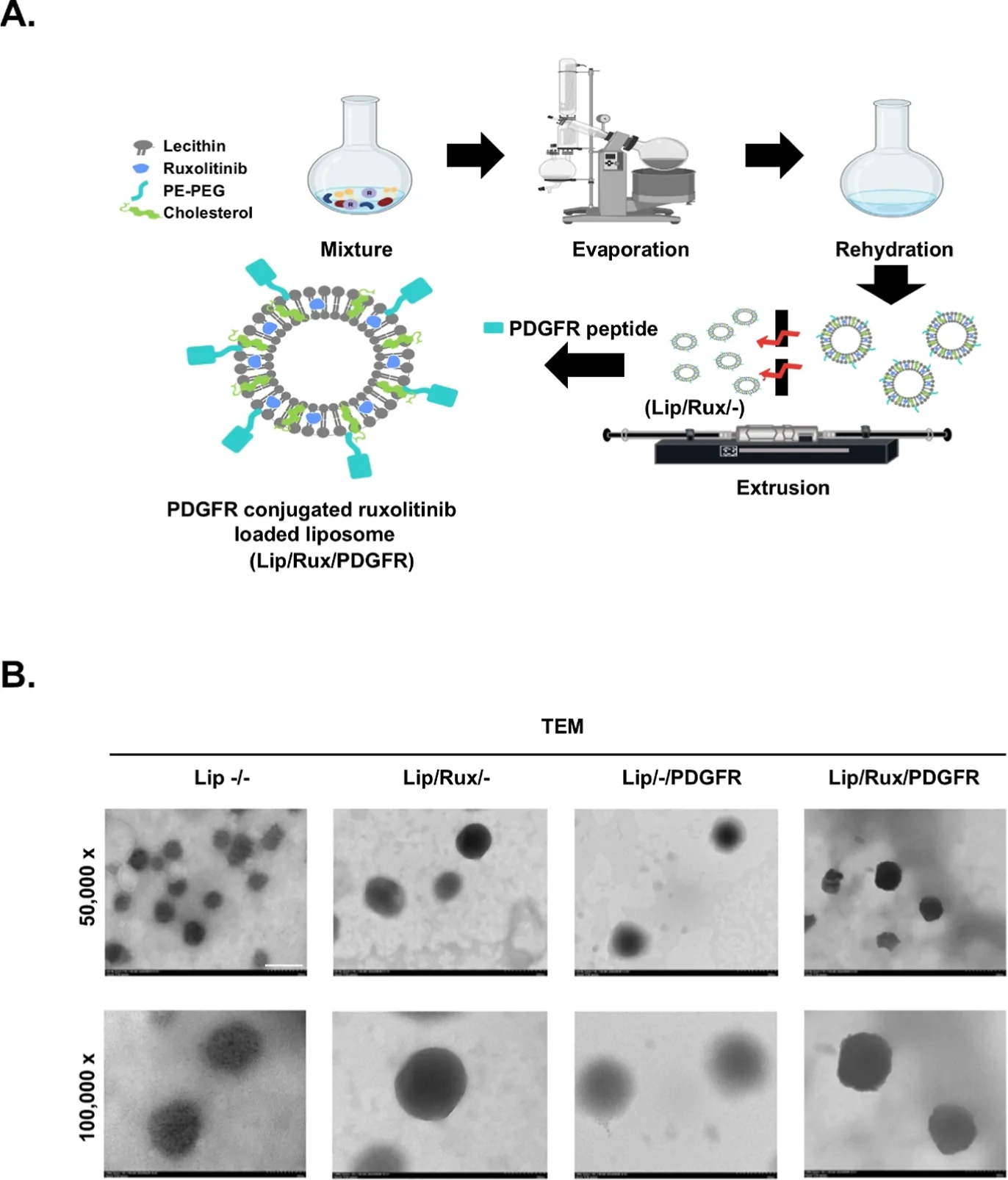
Formulation and morphology of Lip/Rux/PDGFR. (A) Lip/Rux/PDGFR was formulated using the thin film hydration method. (B) Morphology of the formulated Lip/Rux/PDGFR was observed under transmission electron microscopy (TEM) with magnifications 50,000× and 100,000×; scale bar = 200 nm.

**Table 1:** Characterizations of Lip/Rux/PDGFR Nanoparticles^a^

sample name	size (nm)	zeta potential (mV)	encapsulation efficiency (%)
Lip/–/–	196.47 ± 2.95	–7.57 ± 0.27	-
Lip/Rux/-	176.43 ± 6.83	–3.31 ± 0.69	42.86
Lip/-/PDGFR	196.03 ± 5.84	–9.30 ± 0.39	-
Lip/Rux/PDGFR	175.70 ± 2.55	–2.19 ± 0.52	42.86

a Size and zeta potential were determined using DLS. Abbreviations: nm, nanometer; mV, millivolt.

Stability of liposomal formulations has been investigated and included in the Supporting Information (Figure S1). The liposomes were stored at 4 °C for one month to minimize potential drug degradation during storage. Particle size and zeta potential were evaluated using dynamic light scattering (DLS) at predetermined time points. The results demonstrated that neither the average particle size nor the zeta potential showed any statistically significant changes throughout the one-month storage period, indicating good physicochemical stability of the formulation under the tested conditions.

### 3.2 *In Vitro* Drug Release Study of Lip/Rux

The drug release profiles of Lip/Rux/-. The conditions for the drug release study of Lip/Rux/- were determined from the equilibrium solution of Ruxolitinib in PBS (0.01 M) containing 0.5% TWEEN 80 at pH 7.4, as shown in Figure 2. The release of Ruxolitinib from Lip/Rux/- was significantly prolonged compared with free Rux at pH 7.4. The cumulative drug release from Lip/Rux/- was 35.13 ± 2.42%, 59.74 ± 2.41%, and 72.27 ± 5.12%, while from free Rux, it was 92.72 ± 15.93%, 98.94 ± 14.84%, and 99.16 ± 12.46% at 2, 4, and 6 h, respectively. By the end of the experiment, cumulative drug release from Lip/Rux/- was 92.05 ± 3.63%, while from free Rux, it was 98.71 ± 9.04%. Compared with free Rux, Lip/Rux/- was retarded, revealing that Ruxolitinib encapsulation (Lip/Rux/-) could enhance the prolonged release profile. Therefore, the formulated Lip/Rux/- could be used as a nanocarrier for drug delivery of Ruxolitinib with sustained release.

**Figure 2:**
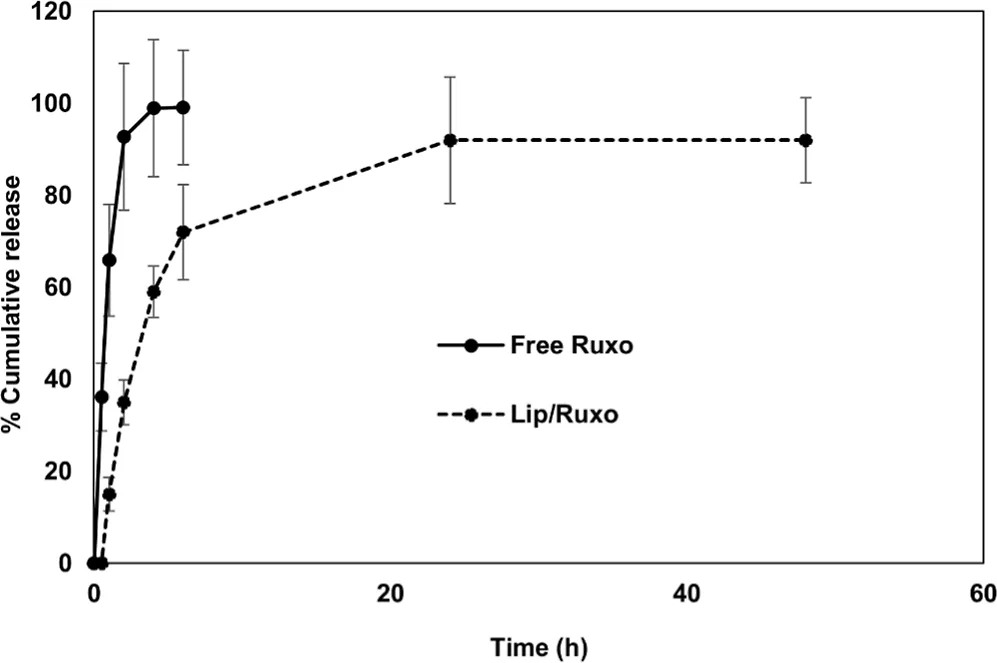
Drug release profile of Lip/Rux. Drug release profiles were assessed using a dialysis bag, molecular weight cutoff of 100 kDa, in phosphate-buffered saline (PBS) pH 7.4, with 0.5% TWEEN 80 at various time points (0–48 h). Data are presented as mean ± SD. The graph represents data from two independent experiments. *p* < 0.05, Student’s *t*-test.

### 3.3 CRC-Derived CM-Induced Activation of Fibroblasts

To mimic the characteristics of CAF, MRC-5 normal human lung fibroblast cells were induced into activated fibroblasts (Act-MRC-5) using CRC-derived CM, as described in Section 2.2. Platelet-derived growth factor alpha receptor-α (PDGFR-α) and alpha-smooth muscle actin (α-SMA) were used as well-known CAFs or activation markers.^10^ Immunofluorescent staining showed that α-SMA expression was increased in the Act-MRC-5 cells compared with MRC-5 cells, as shown in Figure 5A,B. In addition, expression of PDGFRβ was increased in the Act-MRC-5 cells compared with MRC-5 cells, as shown in Supporting Information (Figure S2). This result emphasizes that the CRC-derived CM could successfully induce MRC-5 cells’ fibroblast activation.

### 3.4 Evaluation of Cytotoxicity of Lip/Rux/PDGFR in Fibroblast and CRC Cell Lines

The cytotoxicities of free Rux, Lip/Rux/-, Lip/-/PDGFR, and Lip/Rux/PDGFR in fibroblasts, MRC-5, and Act-MRC-5 were determined using the MTT assay. A subtoxic dose lower than IC_20_ was selected to further study its inactivation of activated fibroblasts (Figure 3A). In, MRC-5 cell, Lip/Rux/PDGFR at a concentration of 10 μM was 80.19 ± 3.5 μM, which had cell viability higher than 80%. At the same concentration, free Rux exhibited fibroblasts’ cell viability at 98.70 ± 2.14%, while that of Lip/Rux/- was 81.24 ± %. A similar result was observed in Act-MRC-5 as shown in Figure 3B. Then concentrations of 5 and 10 μM were selected for inactivating activated fibroblasts in further experiments.

**Figure 3:**
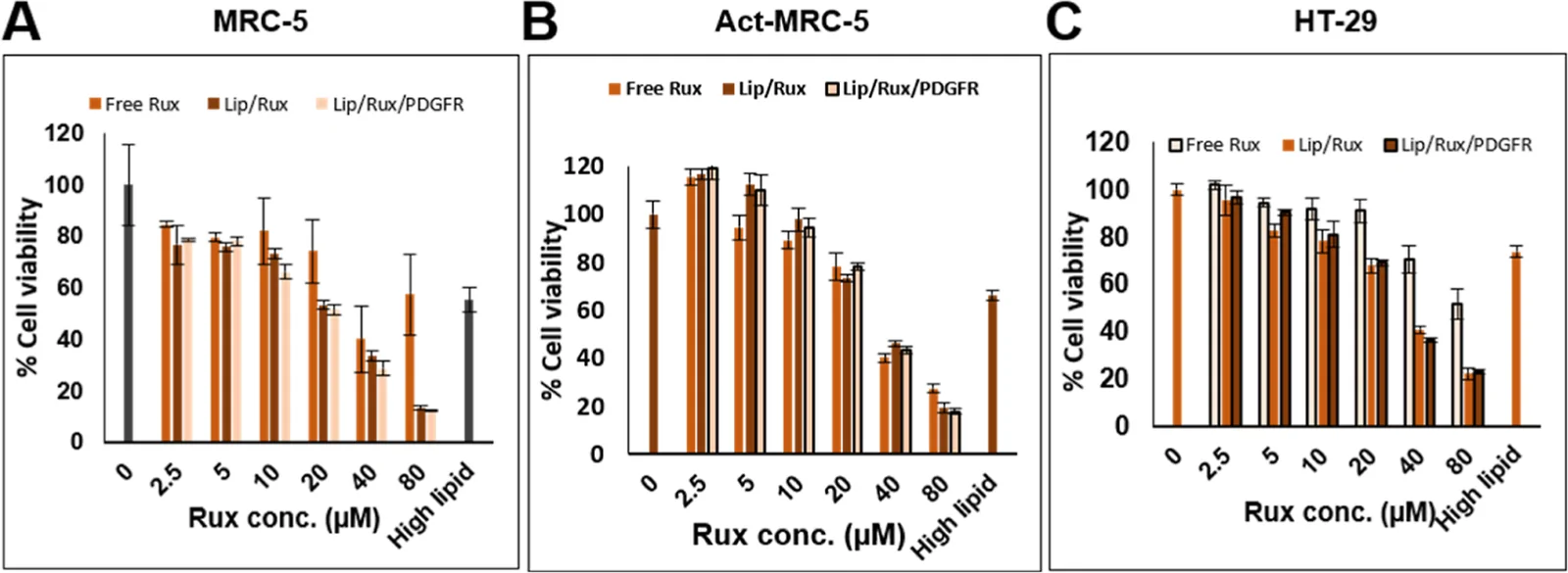
Cytotoxicity of Lip/Rux/PDGFR, free Rux, and Lip/Rux against colorectal cancer and normal human fibroblast cell lines. (A) Cell viability of MRC-5 cells. (B) Act-MRC-5 and (C) HT-29 cells in a monolayer were exposed to Lip/Rux/PDGFR, free Rux, and Lip/Rux for 72 h. The MTT assay was used to analyze cell viability. Data shown are representative of two independent experiments, and error bars represent SD.

For the cytotoxicity of Lip/Rux/PDGFR, free Rux, and Lip/Rux/- in HT-29 cells, a CRC cell line, Lip/Rux/PDGFR, and free Rux reduced HT-29 cells’ viability in a dose-dependent manner, as shown in Figure 3C. In HT-29 cells, cell viability was decreased in dose dependent manners. At 72 h after treatment, cell viabilities of free Rux at concentrations of 10, 20, 40, and 80 μM were 83.15 ± 7.22%, 75.79 ± 6.99%, 52.60 ± 2.37% and 33.32 ± 2.67%, respectively. Cell viabilities of Lip/Rux/- at concentrations of 10, 20, 40, and 80 μM were 97.82 ± 7.21%, 93.06 ± 0.64%, 73.73 ± 4.15% and 20.17 ± 1.55%, respectively. While cell viabilities of Lip/Rux/PDGFR at concentrations of 10, 20, 40, and 80 μM were 96.83 ± 4.44%, 78.22 ± 4.23%, 26.24 ± 2.71%, and 17.80 ± 1.07%, respectively. These results implied that encapsulated Ruxolitinib, both Lip/Rux/PDGFR and Lip/Rux/-, could enhance the cytotoxicity effect of Ruxolitinib in HT-29 cells.

### 3.5 Lip/Rux/PDGFR Enhances Cellular Uptake in Act-MRC-5 Cells

The cellular uptake of Dil-loaded liposomes (Dil/Lip/-) and PDGFR-conjugated liposomes (Dil/Lip/PDGFR) was evaluated in Act-MRC-5 cells for 4 h by using fluorescent microscopy. The PDGFR-specific peptide is a specific peptide that binds to PDGFR, a receptor expressed on CAFs, as shown in Supporting Information (Figure S2).

To further assess the targeting capability of Lip/PDGFR, we evaluated cellular uptake in cell lines with different PDGFR expression levels. Cellular internalization of Lip/PDGFR was evaluated in cell lines with different PDGFR expression levels, including MRC-5 and HT-29 cells (low PDGFR expression) and Act-MRC-5 cells (high PDGFR expression), as shown in Supporting Information (Figure S2). The uptake of targeted and nontargeted liposomes was compared across all three cell lines. An increased cellular uptake of Lip/PDGFR was observed in Act-MRC-5 cells, which exhibit high PDGFR expression at 4 h after treatment, as shown in Figure 4A,B. In contrast, no apparent difference in uptake between targeted and nontargeted liposomes was detected in MRC-5 and HT-29 cells, both of which express low levels of PDGFR as shown in Figure 4C–F, respectively. These results suggest that Lip/PDGFR may enhance cellular uptake in a manner that is associated with PDGFR expression levels. However, the present data do not conclusively establish receptor-mediated targeting, and further studies are required to clarify the underlying mechanism.

**Figure 4:**
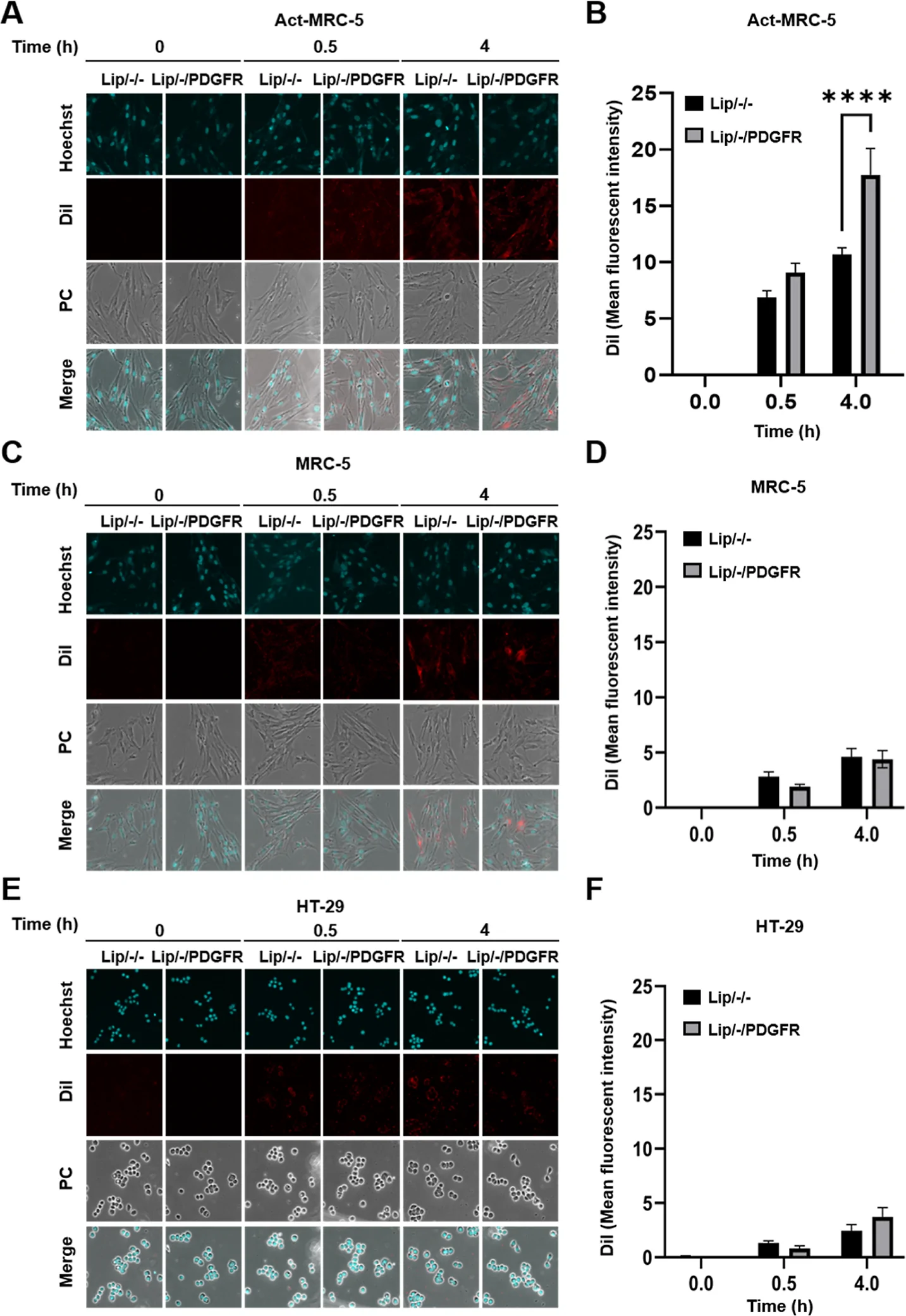
Cellular uptake of Dil-loaded liposomes (Dil/Lip/-) and PDGFR-conjugated liposomes (Dil/Lip/PDGFR) was evaluated in Act-MRC-5 (A,B), MRC-5 (C,D), and HT-29 (E,F) cells by fluorescent microscope (A, C, E) and fluorescent image (B, D, F). The fluorescence intensity was quantified by ImageJ software and plotted in the bar chart as mean ± SD. Statistical analysis was calculated and analyzed using an independent *t*-test (****p < 0.001).

### 3.6 Inactivation of the Activated Fibroblast by Lip/Rux/PDGFR

To assess whether Lip/Rux/PDGFR could modulate the activated fibroblast phenotype, Act-MRC-5 cells were treated with Lip/Rux/PDGFR, free Rux, and Lip/Rux/- for 72 h. α-SMA expression was investigated using immunofluorescent staining. Decreased α-SMA expressions were found in both Lip/Rux/PDGFR- and free-Rux-treated Act-MRC-5 cells compared with the untreated Act-MRC-5 cells, whereas the Lip/–/– -treated cells were unaffected (Figure 5A,B). The decreased α-SMA expression observed following treatment with free Rux, Lip/Rux/-, and Lip/Rux/PDGFR suggests that these formulations may modulate the activated fibroblast phenotype.

**Figure 5:**
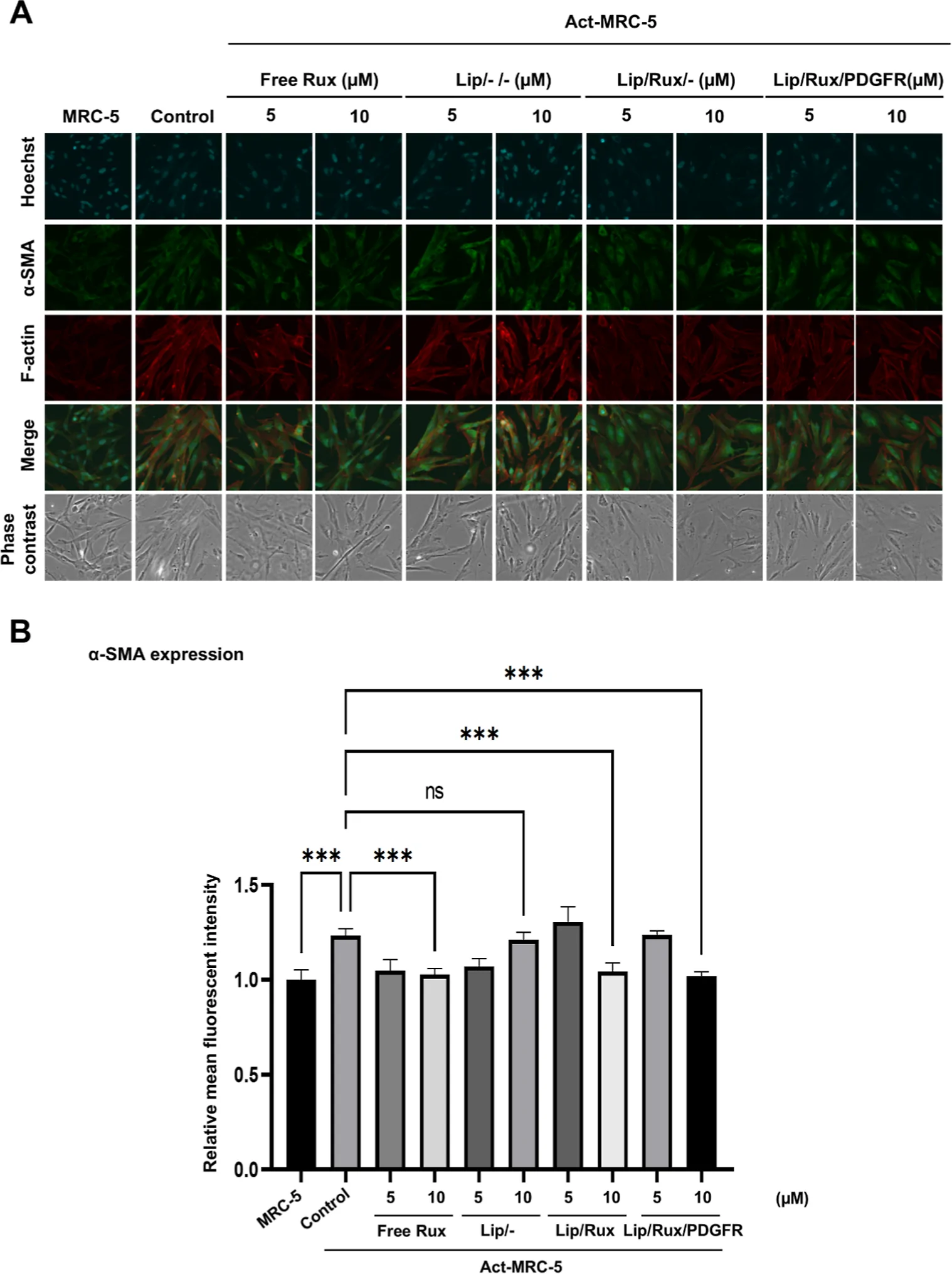
Lip/Rux/PDGFR inhibits activated fibroblasts. The MRC-5 cells were activated with HT-29 cells derived from conditioned medium to become Act-MRC-5 activated fibroblasts. Act-MRC-5 cells were treated with Lip/Rux/PDGFR, free Rux, and Lip/Rux at 5 and 10 μM concentrations for 72 h. (A) α-SMA expression in Act-MRC-5-treated cells was determined by immunofluorescent staining. (B) Quantitative analysis of α-SMA expression (***p < 0.001), one-way ANOVA followed by Tukey’s multiple comparisons test.

### 3.7 Effect of Lip/Rux/PDGFR on Drug Sensitivity of 3D Tumor Spheroids (Monoculture Model)

CRC tumor spheroids were established for testing in both monoculture and coculture conditions and treated with Rux, Lip/Rux/-, or Lip/Rux/PDGFR at 5 and 10 μM in combination with 5-FU at concentrations of 1 and 5 μM. Tumor spheroids’ cell viability was evaluated using the ATPlite luminescence assay. The cell viability of monocultured HT-29 tumor spheroids of 5-FU-treated cells decreased in a dose-dependent manner (Figure 6A,B). Cell viabilities of 5-FU treated HT-29 spheroid at concentrations of 1 and 5 μM was 83.9 ± 5.6% and 62.7 ± 4.7%, respectively. This result showed that 5-FU inhibited cell viability of HT-29 tumor spheroids in a dose-dependent manner.

**Figure 6:**
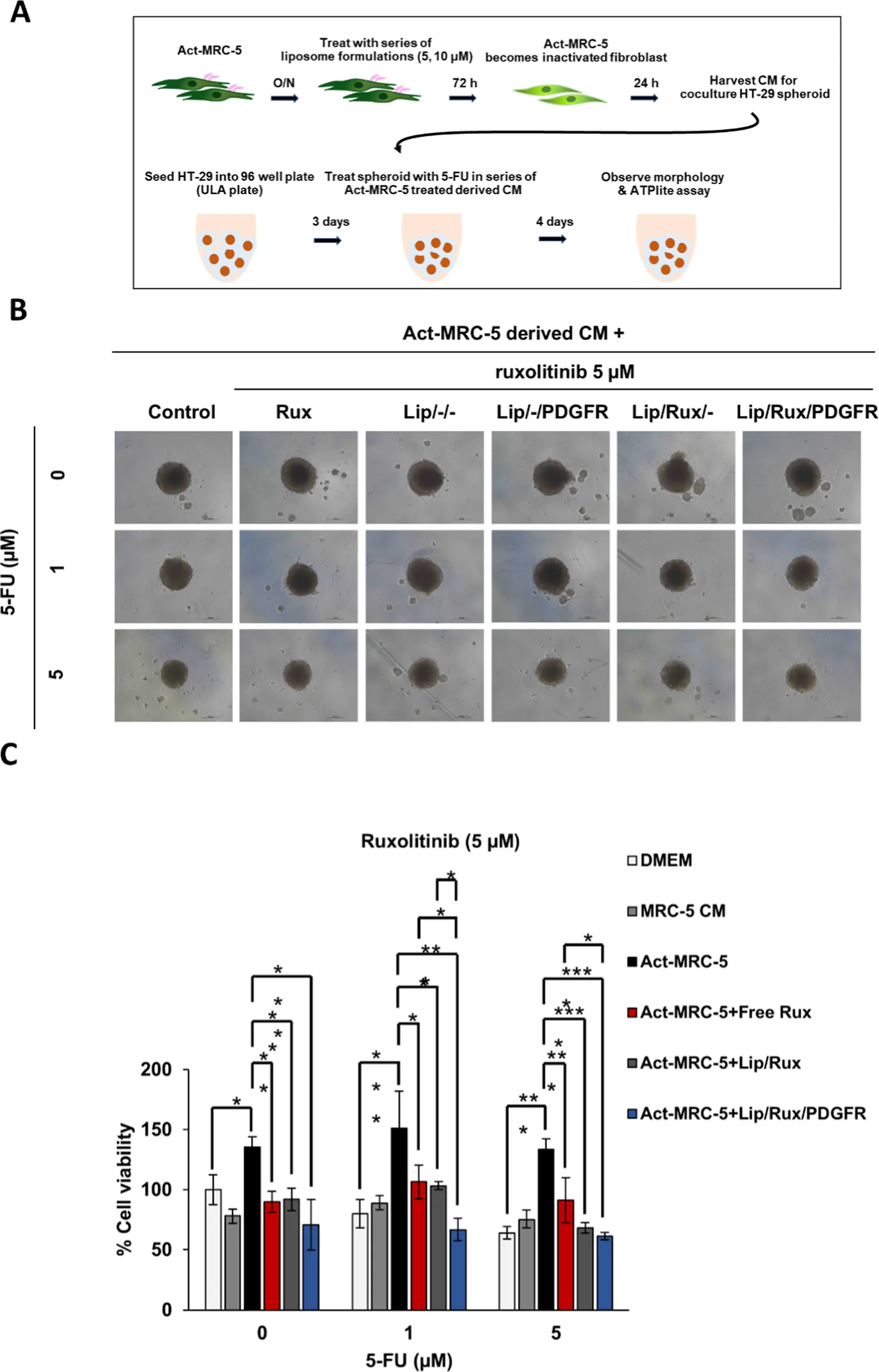
Lip/Rux/PDGFR enhances 5-FU sensitivity in monocultured 3D tumor spheroids. (A) Three-day-old spheroids were treated with free Rux, Lip/Rux, or Lip/Rux/PDGFR at a concentration of 5 μM with or without 5-FU at various concentrations (0, 1, 5 μM) for 96 h. (B) Morphology of the tumor spheroid was observed under a microscope at 10 magnifications. Scale bar = 100 μm. (C) Cell viability of monocultured tumor spheroids was determined using the ATPlite luminescence assay.

In combination of 5-FU at a concentration of 1 μM, cell viability of Rux, Lip/Rux/-, and Lip/Rux/PDGFR (5 μM) treated HT-29 spheroids was 107.0 ± 8.7%, 86.3 ± 4.8%, and 64.6 ± 2.7%, respectively. While in combination with 5-FU at concentrations of 5 μM, treated HT-29 spheroids were 79.6 ± 3.6%, 70.4 ± 14.5%, and 47.4 ± 8.1%. These results revealed that in combination with 5-FU, Lip/Rux/PDGFR showed strongest effect on inhibiting cell viability of HT-29 tumor spheroid in a dose-dependent manner. These results suggested that Lip/Rux/PDGFR enhances 5-FU sensitivity in HT-29 tumor spheroids in the monoculture model.

### 3.8 Effect of Lip/Rux/PDGFR on Drug Sensitivity of 3D Tumor Spheroids (Coculture Model)

Several studies demonstrated that the tumor microenvironment, especially CAFs, can confer growth benefits and induce drug resistance in tumor cells.^34^ To mimic the tumor microenvironment of the tumor, HT-29 tumor spheroids were cocultured with the Act-MRC-5-derived conditioned medium. The tumor spheroids were treated with a series of Rux formulations at concentrations of 5 and 10 μM Ruxolitinib, as shown in Figures 6A and 7A respectively. The morphology of tumor spheroids and cell viability of the tumor spheroids are shown in Figures 6B and 7B. Cell viability of the cocultured-tumor spheroids was strongly reduced in Lip/Rux/PDGFR-treated group compared to other treatment groups, Figures 6C and 7C. Hence, this finding suggested that Lip/Rux/PDGFR is a potent formulation for inhibiting tumor spheroid growth.

**Figure 7:**
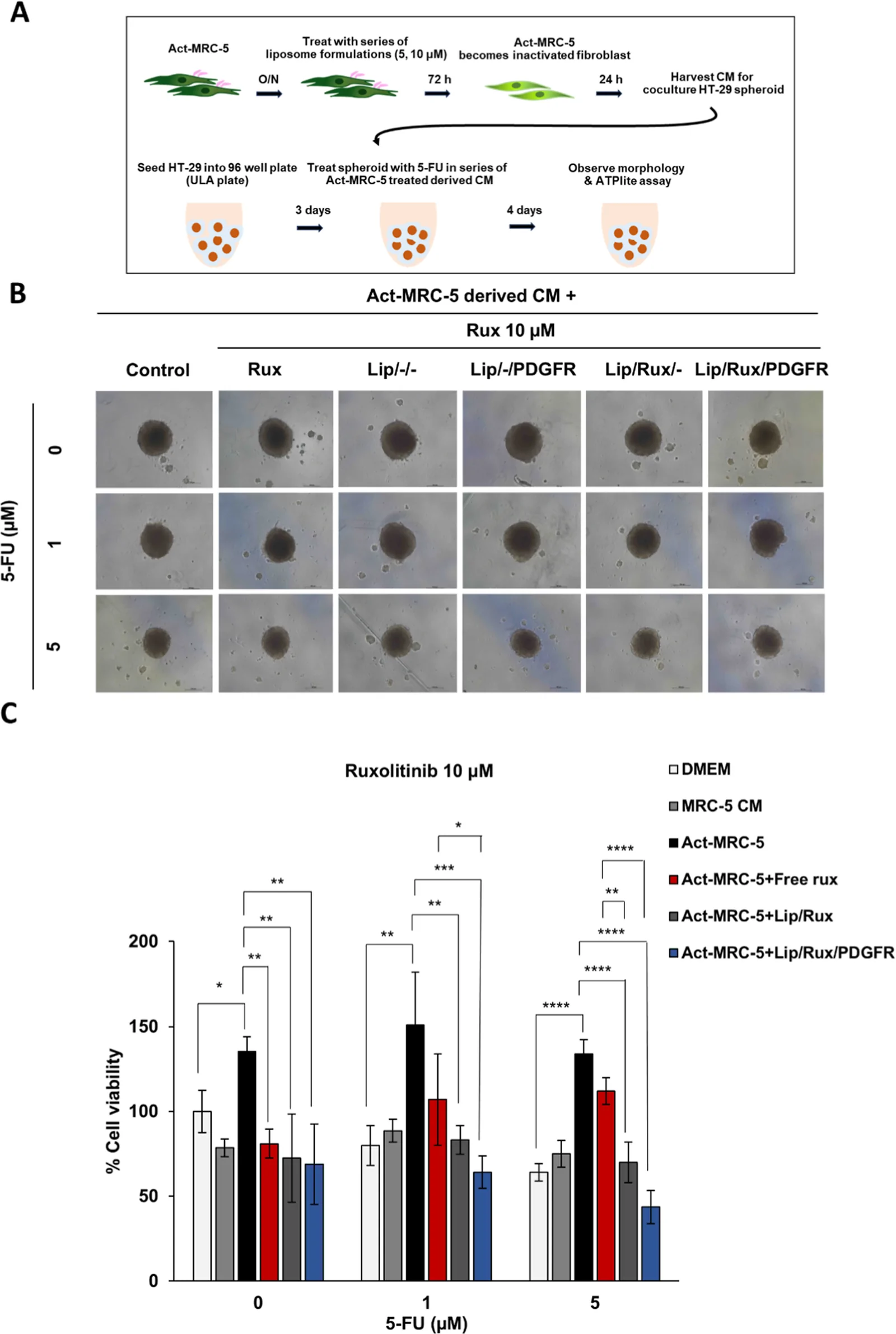
Lip/Rux/PDGFR enhances 5-FU sensitivity in monocultured 3D tumor spheroids. (A) Three-day-old spheroids were treated with free Rux, Lip/Rux, or Lip/Rux/PDGFR at a concentration of 10 μM with or without 5-FU at various concentrations (0, 1, 5 μM) for 96 h. (B) Morphology of the tumor spheroid was observed under a microscope at 10 magnifications. Scale bar = 100 μm. (C) Cell viability of monocultured tumor spheroids was determined using the ATPlite luminescence assay. The data are mean ± SD from the representative experiments. Significant differences were calculated and analyzed using one-way ANOVA, followed by Tukey’s test. *p < 0.05, ***p < 0.001, and ***p < 0.0001.

The tumor spheroids were treated with 5-FU at concentrations of 1 and 5 μM. Cell viability of the spheroid was analyzed based on ATP determination. Cell viability of 5-FU-treated spheroids was decreased in a dose-dependent manner (Figure 6B,C). Cell viabilities of 5-FU-treated tumor spheroidss at concentrations of 1 and 5 μM were 79.8 ± 11.7% and 64.1 ± 5.0%, respectively. In the cocultured tumor spheroid, Lip/Rux/PDGFR exhibited a strong inhibitory effect on tumor spheroid viability compared to Rux and Lip/Rux/-, as shown in Figure 6C.

In combination of 5-FU with a concentration of 1 μM, cell viabilities of cocultured HT-29 spheroids (with Act-MRC-5-derived CM + Rux, Lip/Rux/-, and Lip/Rux/PDGFR) (5 μM) were 106.4 ± 13.9%, 103.2 ± 3.2%, and 66.7 ± 9.4%, respectively. While in combination with 5-FU at concentrations of 5 μM, cell viability was 91.2 ± 18.9%, 68.2 ± 4.5%, and 61.1 ± 3.1%, respectively, as shown in Figure 6B,C. In combination with 5-FU at concentrations of 10 μM, cell viability was 58.1 ± 4.4%, 47.1 ± 6.4%, and 40.7 ± 7.7%, respectively, as shown in Figure 7B,C. These results revealed that in combination with 5-FU, Lip/Rux/PDGFR showed the strongest effect on inhibiting cell viability of HT-29 tumor spheroids in a dose-dependent manner. These results suggested that Lip/Rux/PDGFR enhances 5-FU sensitivity in the cocultured HT-29 tumor spheroid model. Current results correspond with the previous report, which revealed that a combination of Ruxolitinib and vorinostat, an anticancer drug, in a coculturing model between cancer cells and human mesenchymal stem cells could enhance sensitivity of the vorinostat.^35^

### 3.9 Effect of Lip/Rux/PDGFR-Treated Act-MRC-5 Cells on Tumor Spheroid Invasion of CRC Cells

To mimic the environment of a solid tumor, a tumor spheroid was generated to study CRC cell invasion. Several studies revealed that the CAFs, or activated fibroblasts, promote aggressiveness, including the invasive ability of cancer cells.^7,14^ HT-29 tumor spheroids were cocultured with conditioned medium (CM) derived from Act-MRC-5 cells following treatment with various Rux formulations (5 and 10 μM). The resulting CM was used to investigate the impact of treatment-modulated fibroblast-associated factors on the invasive behavior of CRC tumor spheroids (Figures 8 and 9). Increased invasive ability of tumor spheroids was observed when cocultured with activated Act-MRC-5-derived CM compared with the control cells; Figures 8 and 9 show tumor spheroids treated with MRC-5 CM. These results suggest that factors derived from activated fibroblasts may contribute to the enhanced invasiveness of CRC cells. Our results are similar to those of previous studies, which demonstrated that activated fibroblasts or CAFs enhance the invasive ability of several cancer cell types.^36^ As Lip/Rux/PDGFR treatment reduced α-SMA expression in Act-MRC-5 cells, its effect on tumor spheroid invasion was subsequently evaluated. Lip/Rux/PDGFR-treated Act-MRC-5-derived CM (Lip/Rux/PDGFR/Act-MRC-5 CM) showed significantly lower invasive ability in HT-29 spheroid cells compared with the Act-MRC-5 CM. In addition, compared with free-Rux-treated Act-MRC-5, the Lip/Rux/PDGFR-treated cells had a markedly stronger effect on inhibiting HT-29 spheroid invasion. These findings suggest that Lip/Rux/PDGFR treatment may modulate fibroblast-associated factors in conditioned medium, resulting in reduced invasion of HT-29 spheroids. These results suggest that Lip/Rux/PDGFR could represent an alternative approach for reducing cancer cell invasion through modulation of fibroblast-associated tumor–stroma interactions within the TME.

**Figure 8:**
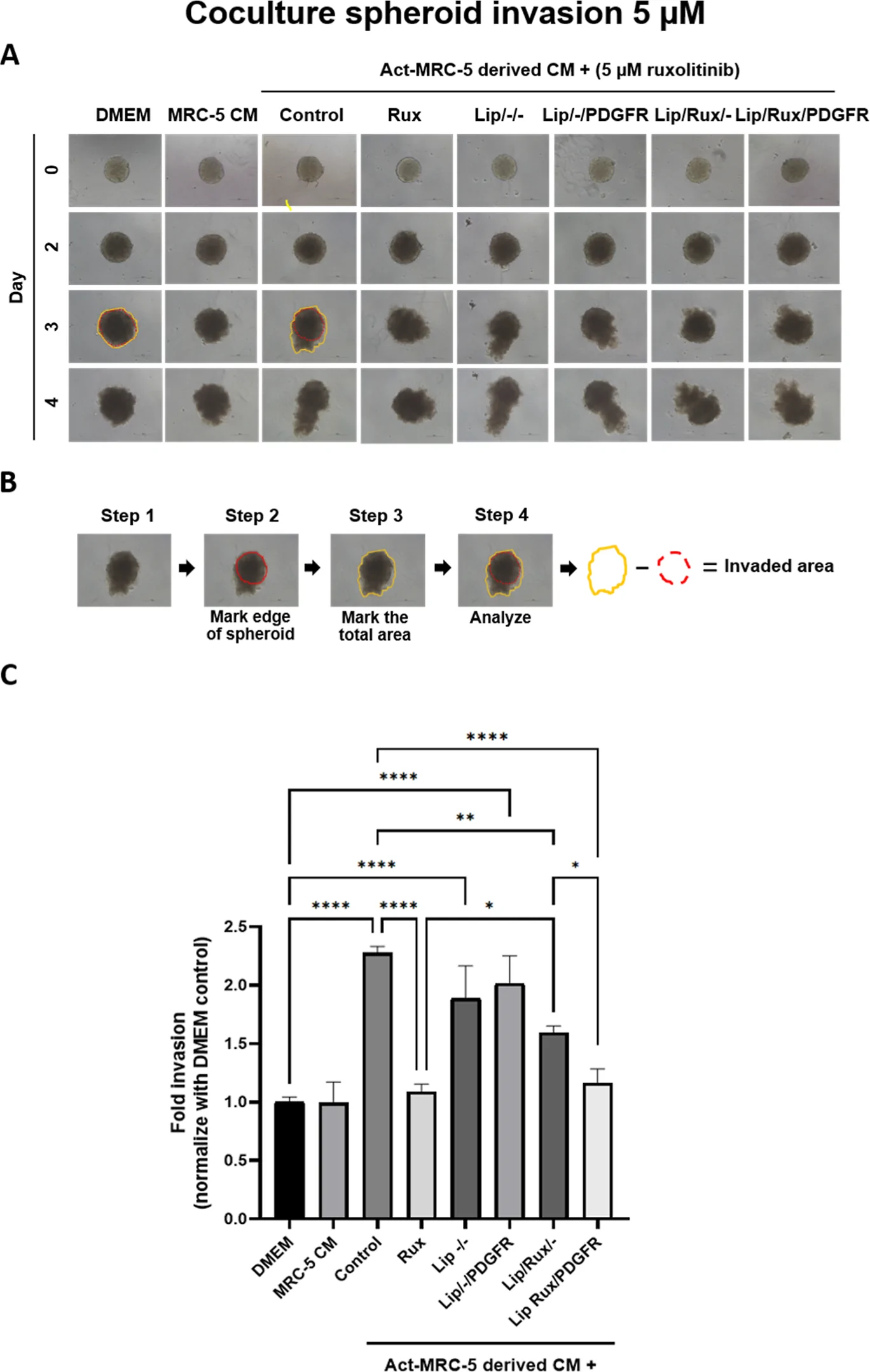
Lip/Rux/PDGFR-treated Act-MRC-5-derived CM (Lip/Rux/PDGFR/Act-MRC-5 CM) treatment alleviated the cancer cell invasion-promoting activity of activated fibroblasts in the 3D tumor spheroid invasion assay. The HT-29 tumor spheroids were established. Three-day-old spheroids were embedded in Matrigel to explore the invasion ability of the tumor spheroid. Then, free Rux, Lip/-, Lip/PDGFR, Lip/Rux, or Lip/Rux/PDGFR-treated Act-MRC-5-derived CM (at a Rux concentration of 5 μM) was added into the spheroid for 96 h, and the invaded area was examined. (A) Morphology of HT-29 tumor spheroid invasion observed under an inverted microscope at 10× magnification. (B) Calculation method of invaded area using ImageJ software. (C) Relative invaded area from the edge of the spheroid determined by ImageJ software (untreated control cells = 1). The data are mean ± SD from the representative experiments. Significant differences were calculated and analyzed using one-way ANOVA, followed by Tukey’s test. *p < 0.05, ***p < 0.001, and ***p < 0.0001.

**Figure 9:**
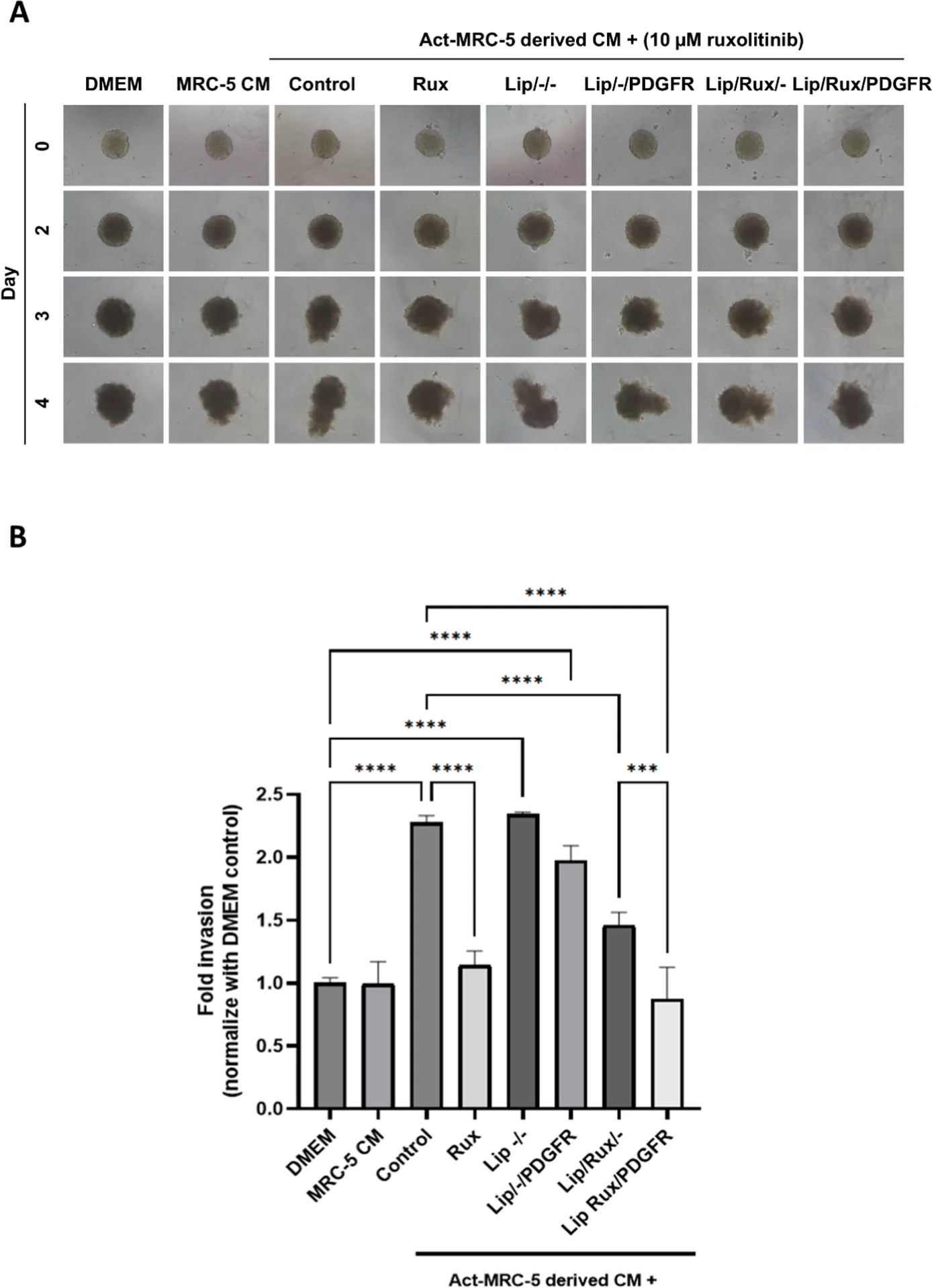
Lip/Rux/PDGFR-treated Act-MRC-5-derived CM (Lip/Rux/PDGFR/Act-MRC-5 CM) treatment alleviated the cancer cell invasion-promoting activity of activated fibroblasts in 3D tumor spheroid invasion assay. The HT-29 tumor spheroids were established. Three-day-old spheroids were embedded in Matrigel to explore the invasion ability of the tumor spheroid. Then, free Rux, Lip/-, Lip/PDGFR, Lip/Rux, or Lip/Rux/PDGFR-treated Act-MRC-5-derived CM (at a Rux concentration of 10 μM) was added into the spheroid for 96 h, and the invaded area was examined. (A) Morphology of HT-29 tumor spheroid invasion observed under an inverted microscope at 10× magnification. (B) Relative invaded area from the edge of the spheroid determined by ImageJ software (untreated control cells = 1). The data are mean ± SD from the representative experiments. Significant differences were calculated and analyzed using one-way ANOVA, followed by Tukey’s test. *p < 0.05, ***p < 0.001, and ***p < 0.0001.

## 4 Conclusions

Lip/Rux/PDGFR enhanced antitumor activity and was associated with reduced α-SMA expression, decreased cancer cell invasion, and enhanced sensitivity to 5-FU in fibroblast-containing models. These findings suggest that Lip/Rux/PDGFR may influence fibroblast-associated tumor–stroma interactions and attenuate tumor-promoting stromal functions. Collectively, the results support the potential of PDGFR-targeted ruxolitinib liposomes as an approach to improve therapeutic responses in colorectal cancer through modulation of the tumor microenvironment. However, the molecular mechanisms responsible for these effects were not directly investigated in this study.

## Supplementary Material



## Notes

Expression of PDGFR in MRC-5, Act-MRC-5, and HT-29 cells determined by immunofluorescent staining and stability of liposomal formulations (PDF)
