# Supplementary material for: PDGFR-Targeted Ruxolitinib Liposomes Modulate Fibroblast-Associated Colorectal Cancer–Stroma Interactions, Reducing Invasion and Enhancing Chemosensitivity
Source: ACS Omega. 2026 Aug 25;11(35):52618–31. doi: 10.1021/acsomega.6c03367 (PMC13563620; doi:10.1021/acsomega.6c03367)
Supplement: Supplementary file 1 [file ao6c03367_si_001.pdf]

PDGFR-Targeted Ruxolitinib Liposomes Modulate Fibroblast-Associated Colorectal Cancer–Stroma Interactions, Reducing Invasion and Enhancing Chemosensitivity

Paweena Dana[1], Anukul Taweechaipaisankul[1,2], Onuma Phoraksa[1], Monthira Rattanatayarom[1], Walailuk Chonniyom[1], Primana Punnakitikashem[3,*], Prattana Tanyapanyachon[1], and Nattika Saengkrit[1,*]

[1] National Nanotechnology Center (NANOTEC), National Science and Technology Development Agency (NSTDA), 111 Thailand Science Park, Phahonyothin Road, Khlong Nueng, Khlong Luang, Pathum Thani 12120, Thailand

[2] Department of Biology, Faculty of Science, Chulalongkorn University, Bangkok 10330, Thailand

[3] Department of Biochemistry, Faculty of Medicine, Siriraj Hospital, Mahidol University, 2 Wanglang Road,

Bangkoknoi, Bangkok 10700, Thailand

* Correspondence: nattika@nanotec.or.th and primana.pun@mahidol.ac.th

Stability of liposomes formulations

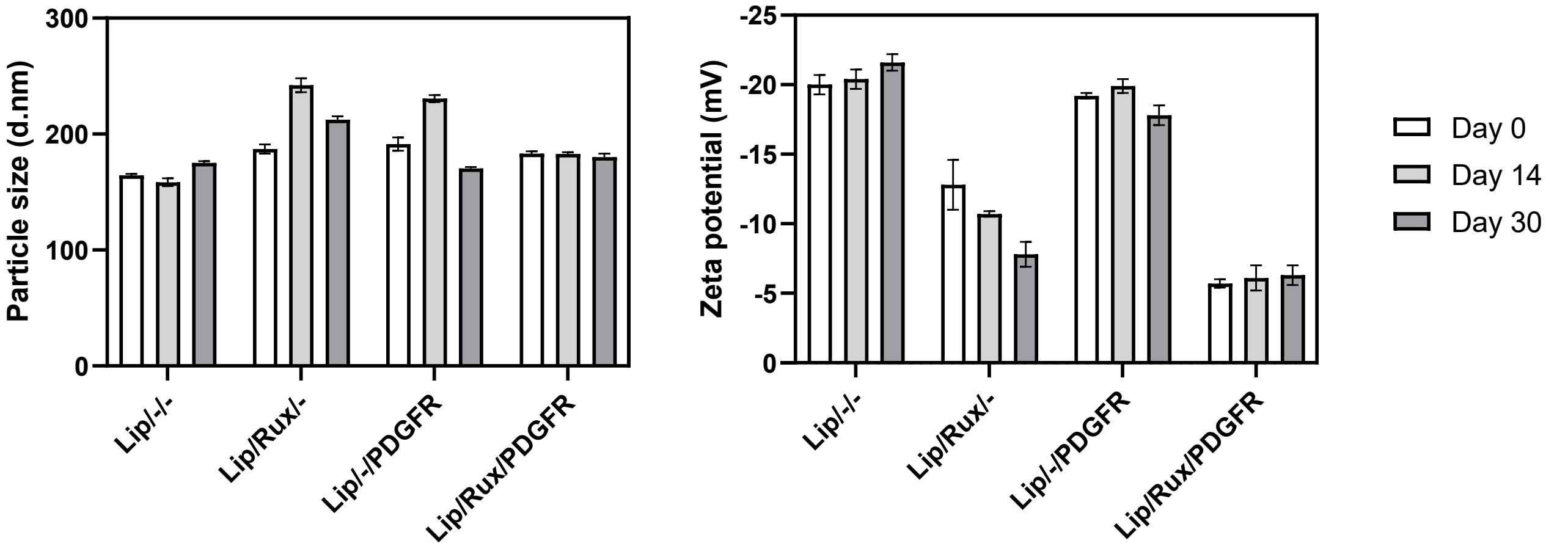

Figure S1: Stability of liposomal formulations was monitored by size and zeta potential analyzed by DLS. (A) Size and zeta potential at the day of formulation, time 0 (B) 30 days after storing at 4 ◦C to avoid drug degradation. Data are presented as mean ± SD.

A.

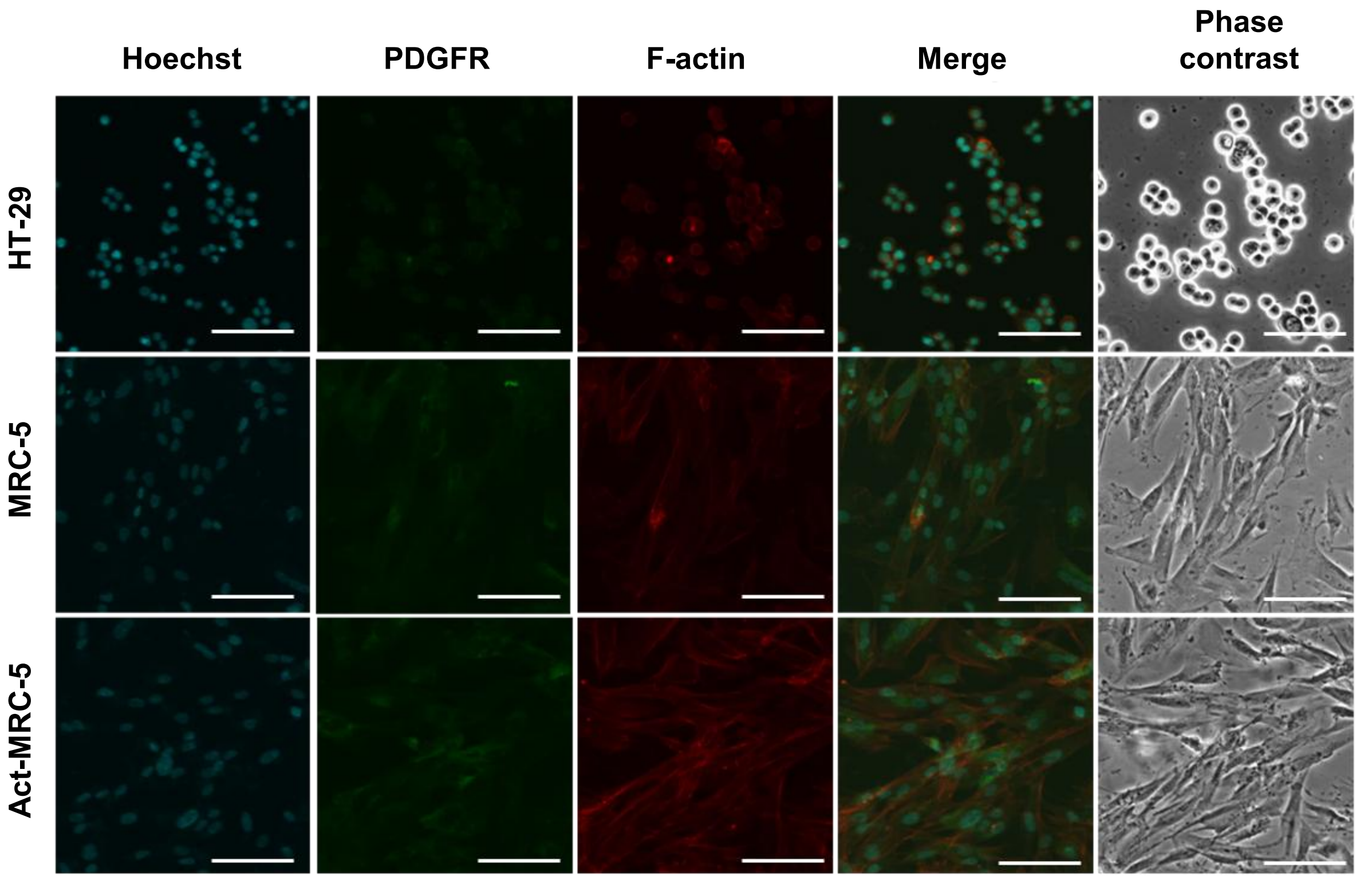

B.

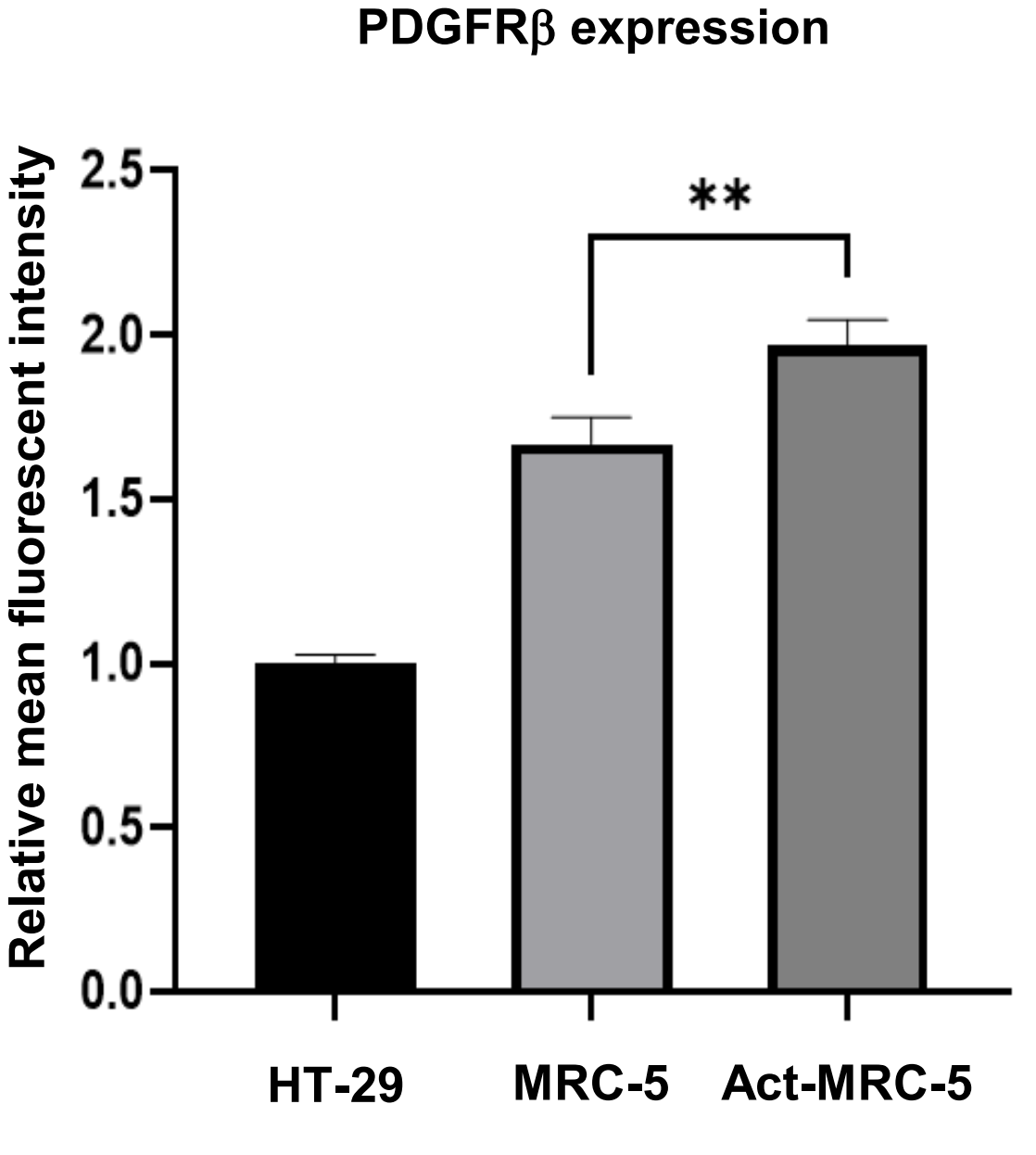

Figure S2: Expression of PDGFRβ in Act-MRC-5 cells. MRC-5 cells were stimulated with HT-29-conditioned medium for 48 h to generate activated fibroblasts (Act-MRC-5). (A) PDGFRβ expression in Act-MRC-5 cells was determined by immunofluorescence staining. (B) Quantitative analysis of PDGFRβ expression. Data are presented as mean ± SD. $^{**}p < 0.01$.
